# Internet-Delivered Cognitive Behavioral Therapy to Treat Insomnia: A Systematic Review and Meta-Analysis

**DOI:** 10.1371/journal.pone.0149139

**Published:** 2016-02-11

**Authors:** Michael Seyffert, Pooja Lagisetty, Jessica Landgraf, Vineet Chopra, Paul N. Pfeiffer, Marisa L. Conte, Mary A. M. Rogers

**Affiliations:** 1 Department of Psychiatry, University of Michigan, Ann Arbor, Michigan, United States of America; 2 Department of Internal Medicine, University of Michigan, Ann Arbor, Michigan, United States of America; 3 Robert Wood Johnson Clinical Scholars Program, University of Michigan, Ann Arbor, Michigan, United States of America; 4 Veterans Administration Center for Clinical Management Research, Veterans Administration Ann Arbor Healthcare System, Ann Arbor, Michigan, United States of America; 5 Health Sciences Library, University of Michigan, Ann Arbor, Michigan, United States of America; Oasi Institute for Research and Prevention of Mental Retardation, ITALY

## Abstract

**Background:**

Insomnia is of major public health importance. While cognitive behavioral therapy is beneficial, in-person treatment is often unavailable. We assessed the effectiveness of internet-delivered cognitive behavioral therapy for insomnia.

**Objectives:**

The primary objectives were to determine whether online cognitive behavioral therapy for insomnia could improve sleep efficiency and reduce the severity of insomnia in adults. Secondary outcomes included sleep quality, total sleep time, time in bed, sleep onset latency, wake time after sleep onset, and number of nocturnal awakenings.

**Data Sources:**

We searched PubMed/MEDLINE, the Cumulative Index to Nursing and Allied Health Literature, PsycInfo, Cochrane Library, Embase, and the Web of Science for randomized trials.

**Methods:**

Studies were eligible if they were randomized controlled trials in adults that reported application of cognitive behavioral therapy for insomnia via internet delivery. Mean differences in improvement in sleep measures were calculated using the Hartung-Knapp-Sidik-Jonkman method for random effects meta-analysis.

**Results:**

We found 15 trials, all utilizing a pretest-posttest randomized control group design. Sleep efficiency was 72% at baseline and improved by 7.2% (95% CI: 5.1%, 9.3%; p<0.001) with internet-delivered cognitive behavioral therapy versus control. Internet-delivered cognitive behavioral therapy resulted in a decrease in the insomnia severity index by 4.3 points (95% CI: -7.1, -1.5; p = 0.017) compared to control. Total sleep time averaged 5.7 hours at baseline and increased by 20 minutes with internet-delivered therapy versus control (95% CI: 9, 31; p = 0.004). The severity of depression decreased by 2.3 points (95% CI: -2.9, -1.7; p = 0.013) in individuals who received internet-delivered cognitive behavioral therapy compared to control. Improvements in sleep efficiency, the insomnia severity index and depression scores with internet-delivered cognitive behavioral therapy were maintained from 4 to 48 weeks after post-treatment assessment. There were no statistically significant differences between sleep efficiency, total sleep time, and insomnia severity index for internet-delivered versus in-person therapy with a trained therapist.

**Conclusion:**

In conclusion, internet-delivered cognitive behavioral therapy is effective in improving sleep in adults with insomnia. Efforts should be made to educate the public and expand access to this therapy. Registration Number, Prospero: CRD42015017622

## Introduction

Insomnia is estimated to affect 9%-15% of the world’s population [1]. Not only is insomnia common, it is persistent over time. In a population-based longitudinal study characterizing the natural history of insomnia, the point prevalence of insomnia was 24% at baseline and, among individuals with insomnia, 74% reported the persistence of this disorder for at least one year [2]. In addition, chronic insomnia disorder is associated with other chronic conditions such as obesity, diabetes, hypertension, cardiovascular disease, anxiety and depression [1,3,4]. Notably, the relationship between insomnia and depression is bi-directional; individuals with insomnia are at significantly higher risk of developing depression, and persons with depression are at higher risk of developing insomnia [5].

Diagnostic criteria for chronic insomnia disorder include problems with initiating or maintaining sleep for at least 3 months (occurring at least 3 times per week) despite opportunities to sleep, as well as impairment in daytime functioning [6]. These criteria were recently updated in the third edition of the International Classification of Sleep Disorders (ICSD-3) [6].

Treatment of insomnia predominantly occurs in primary care and outpatient mental health settings, with cognitive behavioral therapy for insomnia (CBTI) often offered as a first-line therapy in adults [7]. The American Academy of Sleep Medicine recommends CBTI for chronic primary insomnia disorder with and without comorbid conditions [7]. The United States Preventive Services Task Force found that the net benefit of CBTI was moderate from high quality studies [8]. When administered in-person by a trained therapist, CBTI has been shown to be effective in improving sleep, with clinically meaningful effect sizes [9]. Pharmacologic treatments, such as benzodiazepine-receptor agonists and low dose antidepressants, are also commonly used therapies for insomnia and are sometimes used in patients based on symptom patterns, coexisting diseases, and previous treatment responses [7,10]. Randomized trials comparing medications for insomnia versus CBTI indicate that CBTI yields more durable sleep improvement over the course of time with fewer side effects [11].

Despite successes with face-to-face therapists, CBTI is not easily accessible to all individuals with insomnia. There is a lack of well-trained therapists specializing in CBTI to fill the need, given the millions of individuals who may benefit [1]. To address this issue, some investigators have examined whether CBTI programs could be administered through the Internet as a means for disseminating insomnia treatment to a wider audience [12,13]. Internet-delivered therapies have been previously found to be effective in particular settings, such as in the workplace to reduce anxiety [14], and for disease management in patients with type 2 diabetes [15]. Internet-delivered therapy when guided by a therapist was found to be equivalent to face-to-face CBT for certain psychiatric and somatic disorders [16].

Recently, there have been a number of trials published which evaluated the efficacy of CBTI through internet delivery. A review of self-directed therapies for insomnia concluded that self-directed treatments were efficacious, although the majority of the interventions in this review were delivered through booklets, audiotapes, or videotapes [17]. Evidence from six randomized trials on computerized CBTI yielded results showing mild to moderate improvement in sleep with computerized CBTI, but not all studies included an internet platform [12]. A recent review presented evidence from 11 trials, finding improvement in the severity of insomnia and sleep efficiency, with the duration of treatment and the degree of support as moderators of sleep efficiency [13]. We independently reviewed the evidence from randomized controlled trials regarding CBTI in adults. Our primary hypotheses were that internet-delivered CBTI would improve sleep efficiency and reduce the severity of insomnia in adults when compared to a wait list or CBTI through other delivery methods. While our primary outcomes of interest were sleep efficiency and severity of insomnia, secondary outcomes were sleep quality, total sleep time, time in bed, sleep onset latency, wake time after sleep onset, and number of nocturnal awakenings.

## Methods

### Eligibility criteria

The Preferred Reporting Items for Systematic Reviews and Meta-Analyses (PRISMA) guidelines were used [18] (S1 File). Studies were eligible if they were randomized controlled trials that reported application of CBTI via internet delivery. The primary outcomes of interest were sleep efficiency and severity of insomnia. Secondary outcomes were sleep quality, total sleep time, time in bed, sleep onset latency, wake time after sleep onset, and number of nocturnal awakenings. All comparators to internet-delivered CBTI were included, as reported in the trials. Only trials utilizing a comprehensive package of CBTI were included (i.e., the core elements include sleep education, stimulus control, sleep restriction, relaxation, sleep hygiene, cognitive techniques such as restructuring, reappraisal, visualization, focusing, paradox, mindfulness); those studies that used an abbreviated version or a modified version of CBTI were excluded. We excluded studies involving children less than 16 years of age as reasons for insomnia and treatment in this group are heterogeneous and distinct to adults. Similarly, trials targeting specific patient groups (patients with organic brain disorders, pain, tinnitus or cancer) were excluded as the causes and treatment of insomnia may also be different in these populations.

### Literature search and study selection

Working with a biomedical research librarian, specific search strategies were developed for the following databases: PubMed/MEDLINE via the National Library of Medicine (www.pubmed.gov), the Cumulative Index to Nursing and Allied Health Literature (CINAHL) and PsycInfo via Ebsco, Cochrane Library, Embase, and Web of Science. Search strategies utilized a combination of keywords and controlled vocabularies (eg Medical Subject Headings and Emtree terms), combined with with Boolean operators, to represent the concepts of insomnia, cognitive behavior therapy, web-based interventions and randomized trials. No date or language limits were applied (date of search: September 1, 2015); all search strategies are supplied in S2 File. Abstracts of all retrieved studies were scanned for eligibility. Additionally, references from published systematic reviews and meta-analyses were evaluated by hand. Two reviewers (PL, MR) independently evaluated the full publication of identified studies to determine eligibility. Disagreements between reviewers were resolved by deliberation by a third author (VC).

### Data extraction

Data regarding study design, participant characteristics, treatment arms, sleep measures, treatment duration, baseline measures, post-treatment measures, and follow-up measures were extracted by two abstractors (MS, JL) and checked by another (MR) (S1 Dataset). Study authors were contacted by email to clarify data that were ambiguous.

### Statistical analyses

All randomized trials included both a within-person comparison (before-after) as well as a between-group comparator. The statistical analyses were designed to evaluate both of these effects. First, mean pretest-posttest change in sleep measures within individuals were calculated (i.e., using the before measurement and the immediate post-treatment measurement), as well as the standard deviation using recommended procedures [19]. The correlation coefficient (r) for calculating the standard deviation for grouped paired data was obtained from information within the trials (using the standard deviation of change scores); the mean r was 0.587 (range 0.474, 0.712). This agrees with the median correlation for change from baseline in systematic reviews, as previously reported to be 0.59 [20]. Next, between-group comparisons were made using the intervention (internet-delivered CBTI) versus comparators. Therefore, the primary effect estimate was the mean difference in the within-person change in sleep measure with internet-delivered CBTI and the within-person change in sleep measure with the comparator. This final metric reflects the degree of improvement in sleep measures by using an internet-based approach for delivery versus improvement using another approach. To enhance the interpretability of the final measures, we retained the original units of the sleep measures (e.g., number of minutes slept). There was only one exception; there was variation in the number of questions utilized for the Dysfunctional Belief and Attitudes about Sleep Scale and, therefore, a standardized mean difference was calculated. As a secondary outcome, changes in the severity of depression were calculated when available in the trials.

A wait list control was the most common comparator and was employed first. Secondly, we examined other comparators that involved therapists (group face-to-face, group telehealth, enhanced email contact, phone contact). An additional comparator in the eligible trials was CBTI via printed materials only. For the between-group comparisons, the Hartung-Knapp-Sidik-Jonkman (HKSJ) method for random effects meta-analysis was used because it has been shown to outperform the DerSimonian-Laird method [21,22]. Results were pooled when there were 2 or more trials with a common comparator and common outcomes. Alpha was set at 0.05, 2-tailed.

In secondary analyses, long-term assessments of sleep measures were evaluated for those studies that provided data on extended follow-up (4 weeks to 48 weeks after the immediate post-test assessment). In an additional secondary analysis, the mechanism by which CBTI influences sleep measures was examined by pooling information regarding dysfunctional beliefs (using the Dysfunctional Belief and Attitudes about Sleep scale).

Heterogeneity was evaluated using Cochran's Q test of heterogeneity and the I^2^ statistic, which measures the proportion of inconsistency among studies that cannot be explained by chance. To assess the possibility of publication bias, the Egger regression asymmetry test for publication bias was calculated for the sleep outcomes. Risk of bias across studies was estimated independently by 2 authors (JL, MR) using the Cochrane Collaboration’s tool for assessing risk of bias [23]. This was enhanced by features of the experimental design (concurrent comparator, within-person comparator, type of negative control) and use of intent-to-treat analyses.

Meta-regression was performed to evaluate whether age or gender affected the associations between CBTI and the primary outcomes, sleep efficiency and insomnia severity index. It was also performed for the secondary outcomes: total sleep time, sleep onset latency, and wake time after sleep onset. The Knapp Hartung modification was used to calculate the variance of the estimated coefficients.

Completion rates were assessed in each study arm (internet-delivered CBTI versus wait list control); the numerator contained the number of subjects who completed the sleep diaries and the denominator contained the number of subjects randomized. Completion rates were pooled using a random effects model with Freeman-Tukey double arcsine transformation to stabilize the variance and exact binomial (Clopper-Pearson) 95% confidence intervals.

## Results

### Study Characteristics

**Fig 1:**
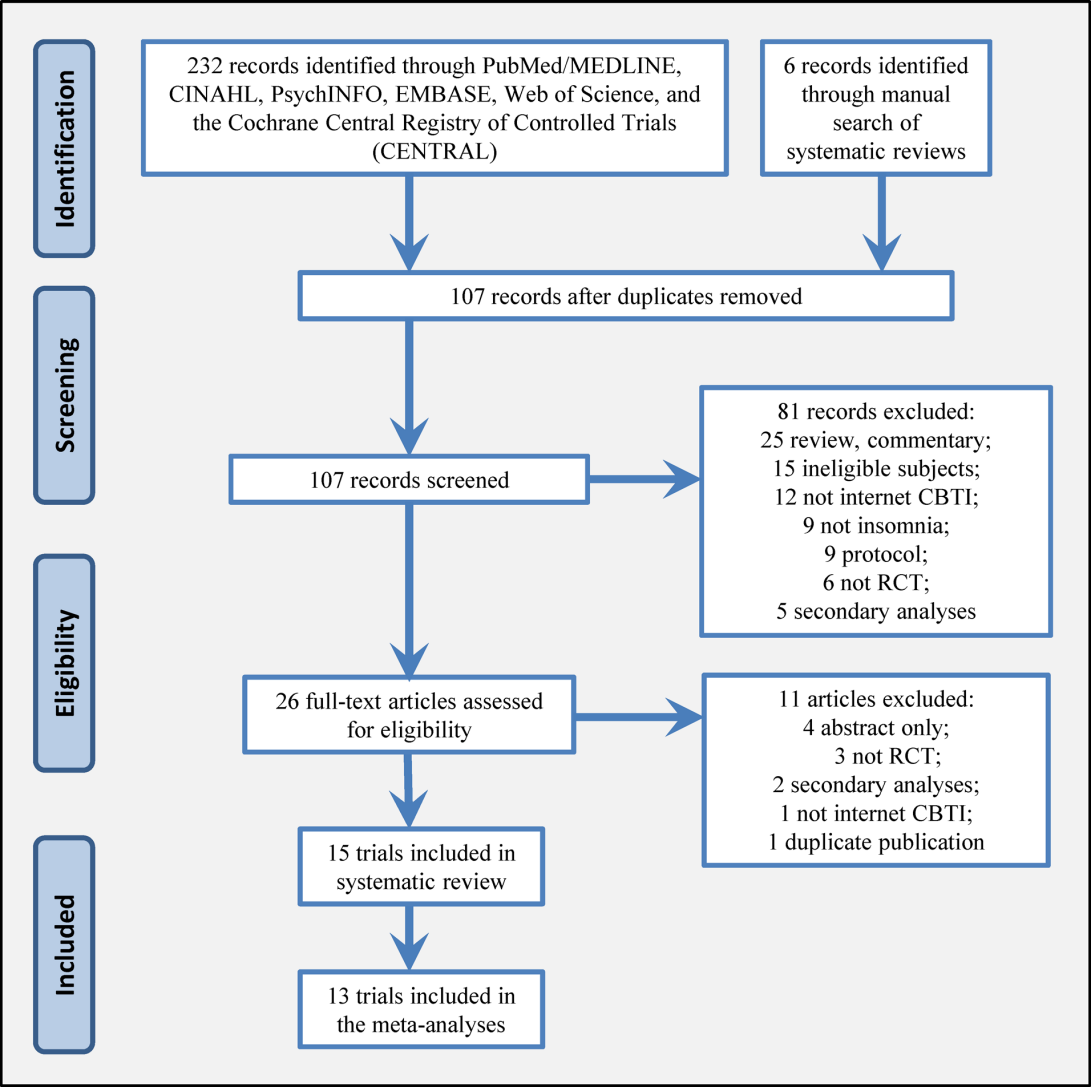
PRISMA Flow Diagram for the Systematic Review of Internet-Delivered Cognitive Behavioral Therapy to Treat Insomnia.

The search retrieved 232 records from electronic databases and 6 records from the manual search (Fig 1). A total of 107 unique abstracts were reviewed and 26 full-text articles were assessed for eligibility. Following review of these articles, 15 randomized trials with a total of 2,392 participants met the criteria for inclusion in this systematic review [24–38]. Of these, 13 trials contained similar comparators which enabled pooling through meta-analysis [25–30, 32–38].

Eleven trials were conducted in Europe (Sweden, United Kingdom, the Netherlands, Germany), while 2 were conducted in Canada, 1 in the United States, and 1 in China (Table 1). Most trials were conducted in community residents while 3 targeted specific populations (teachers, university students, patients with both insomnia and major depression). Most trials were conducted in middle-aged individuals, the majority of whom were women. The presence of psychiatric and other comorbidities in participants occurred in approximately 10% in some trials [30, 31, 36], 30% in one trial [26], approximately 50% in others [28, 35, 38], and 100% in another [24].

**Table 1:** Study Characteristics.

Author	Year	Location	Participants	Number Randomized	Number of Study Arms (Types)	% Female	Age (years)
Blom[24]	2015	Stockholm County, Sweden	Adults with both insomnia and major depression	43	2 (Internet CBTI, Internet CBT for depression)	53%	Mean 48 (CBTI); Mean 46 (CBTD)
Blom[25]	2015	Ostergotland County, Sweden	Adults with insomnia, community residents	48	2 (Internet CBTI, Group-delivered In-Person CBTI)	48%	Mean 56 (CBTI); Mean 53 (GCBT)
Espie[26]	2012	United Kingdom	Adults with insomnia, community residents	164	3 (Internet CBTI, Imagery Relief Therapy, Treatment as Usual)	73%	Mean 51 (CBTI); Mean 47 (IRT); Mean 49 (TAU)
Ho[27]	2014	Hong Kong	Adults with insomnia, community residents	312	3 (Internet CBTI & phone support, Internet CBTI & no support, Wait list)	71%	Mean 37 (CBTI support); Mean 39 (CBTI); Mean 40 (WL)
Holmqvist[28]	2014	Canada	Adult residents of rural province with insomnia	73	2 (Internet CBTI, Telehealth CBTI)	75%	Not stated
Kaldo[29]	2015	Stockholm County, Sweden	Adults with insomnia, community residents	148	2 (Internet CBTI, Internet control)	78%	Mean 47 (CBTI); Mean 49 (Control)
Lancee[30]	2012	The Netherlands	Adults with insomnia, community residents	623	3 (Internet CBTI, Pen & Pencil CBTI, Wait List)	70%	Mean 52 (Internet CBTI); Mean 51 (Pen CBTI); Mean 52 (WL)
Lancee[31]	2013	The Netherlands	Adults with insomnia, community residents	262	2 (Internet CBTI & email support, Internet CBTI & no support)	75%	Mean 49 (CBTI Support); Mean 47 (CBTI no support)
Lancee[32]	2015	The Netherlands	Adults with insomnia, community residents	63	2 (Internet CBTI, Wait List)	79%	Mean 47 (CBTI); Mean 50 (WL)
Morris[33]	2015	United Kingdom	University students	138	3 (Internet CBTI, Internet CBT for anxiety, Wait List)	67%	Mean 21 (Internet CBTI); Mean 21 (Anxiety CBT); Mean 20 (WL)
Ritterband[34]	2009	Virginia, United States	Adults with insomnia, community residents	45	2 (Internet CBTI, Wait List)	76%	Mean 45 (CBTI); Mean 45 (WL)
Strom[35]	2004	Sweden	Adults with insomnia, community residents	109	2 (Internet CBTI, Wait List)	65%	Mean 46 (CBTI); Mean 44 (WL)
Thiart[36]	2015	Germany	Adult teachers with insomnia	128	2 (Internet CBTI, Wait List)	74%	Mean 48 (CBTI); Mean 48 (WL)
van Straten[37]	2014	The Netherlands	Adults with insomnia, community residents	118	2 (Internet CBTI, Wait List)	70%	Mean 49 (CBTI); Mean 50 (WL)
Vincent[38]	2009	Canada	Adults with insomnia, community residents	118	2 (Internet CBTI, Wait List)	67%	Not stated

Eleven trials included 2 study arms (i.e., internet-delivered CBTI vs. comparator) while four trials included 3 study arms; the comparators are listed in Table 1. Twelve of the trials included additional information regarding long-term follow-up, ranging from 4-weeks to 1-year measurements after completion of the post-test assessment [24–32, 36–38].

The most commonly reported sleep measure outcomes were sleep efficiency and total sleep time (Table 2). Other common outcomes included sleep onset latency, wake time after sleep onset, sleep quality, insomnia severity index and the number of awakenings. Although all trials utilized internet-delivered CBTI and contained the basic elements of this therapy, there were slight differences in the duration of the programs across the 15 trials. For example, internet CBTI was administered through a 6-week program in 8 trials [26–28,30,31,33,36,37], whereas 2 trials used a 5-week approach [35,38], 3 an 8-week approach [25,29,32], and 2 a 9-week approach [24,34].

**Table 2:** Description of Sleep Measures*.

Measure	Abbreviation	Minimum value	Maximum value	Interpretation
Total Sleep Time	TST	0 hours	24 hours	A count of the total amount of time (in minutes or hours) slept in a 24-hour period.
Time In Bed	TIB	0 hours	24 hours	A count of the total time (in minutes or hours) spent in bed in a 24-hour period.
Sleep Efficiency	SE	0%	100%	The percentage of "Time in Bed" that is spent sleeping (Total Sleep Time). Value ≥85% indicates clinically normal sleep and not suggestive of insomnia.
Wake Time After Sleep Onset	WASO	0 minutes		A count of the total minutes of wakefulness after the onset of sleep.
Sleep Onset Latency	SOL	0 minutes		The time (minutes) it takes to fall asleep. Calculated as the difference between bedtime (lights out) to when sleep begins.
Insomnia Severity Index	ISI	0 score	28 score	Sum of 7 sleep-related questions (using 5-point likert scale). 0–7 indicates no clinically significant insomnia, 8–14 subthreshold insomnia, 15–21 insomnia of moderate severity, 22–28 severe insomnia.
Pittsburgh Sleep Quality Index	PSQI	0 score	21 score	Sum of 7 sleep-related questions. Increasing value indicates poorer sleep quality. PQSI >5 is associated with poor sleep quality.
Nocturnal Awakenings	NWAK	0 times		Number of times a person awoke during the night.
Sleep Quality	SQ	0 score	4 score	Measured on a 5-point scale from very poor to very good.

* Time measures were recorded using sleep diaries.

### Internet CBTI versus Wait List

**Fig 2:**
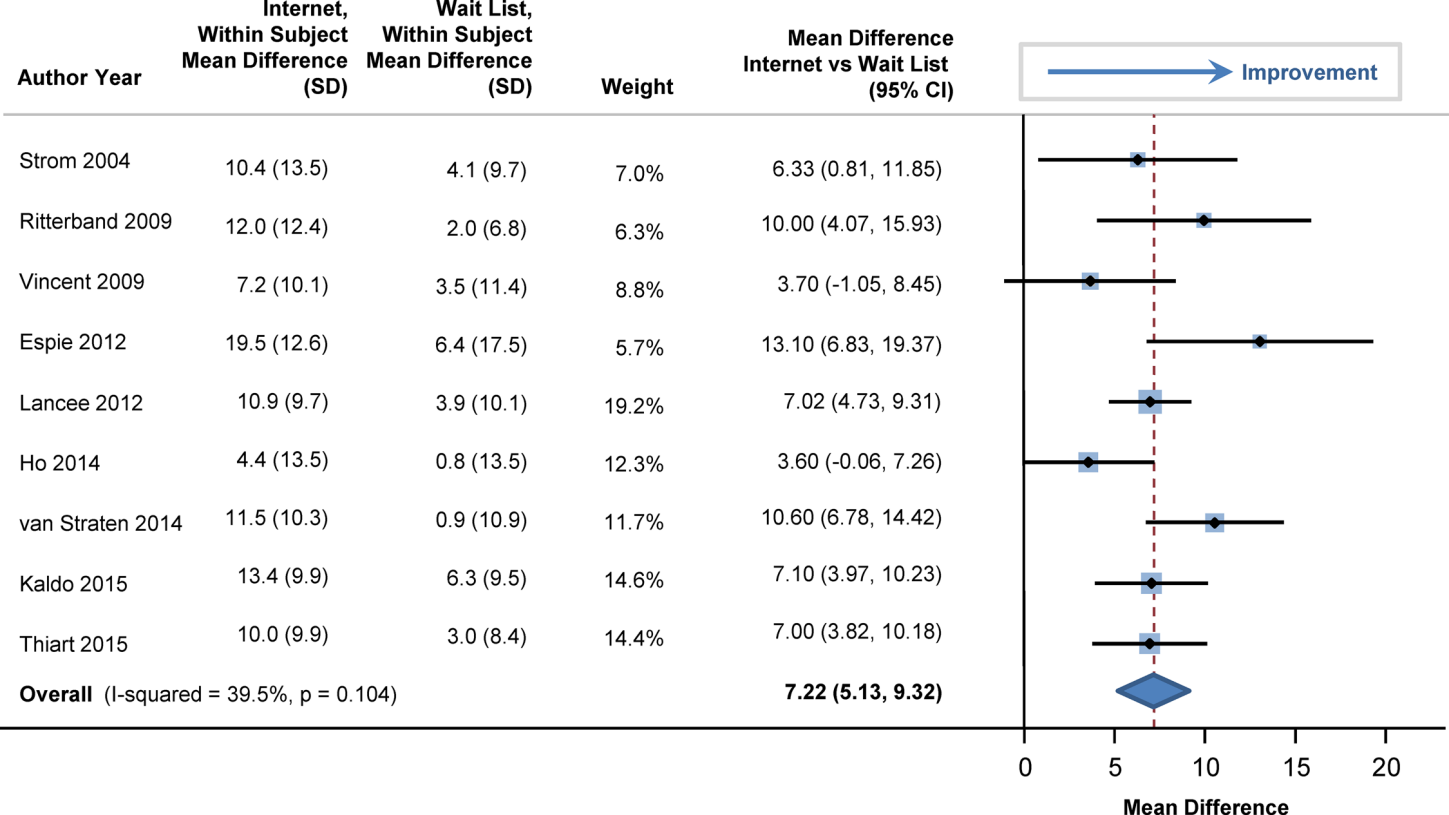
Mean Differences in Sleep Efficiency between Internet-Delivered Cognitive Behavioral Therapy for Insomnia and Wait List.

**Fig 3:**
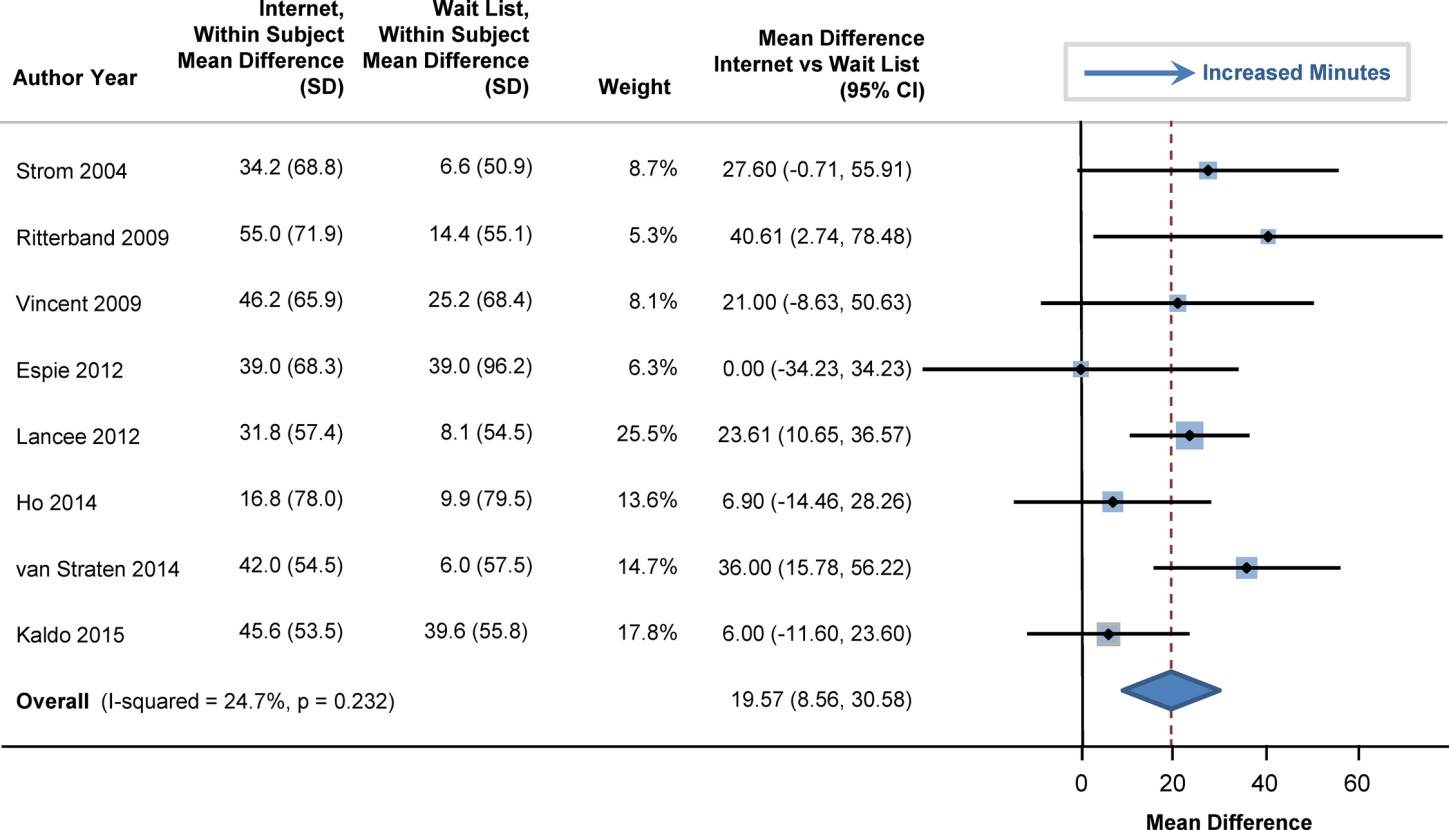
Mean Differences in Total Sleep Time between Internet-Delivered Cognitive Behavioral Therapy for Insomnia and Wait List.

**Fig 4:**
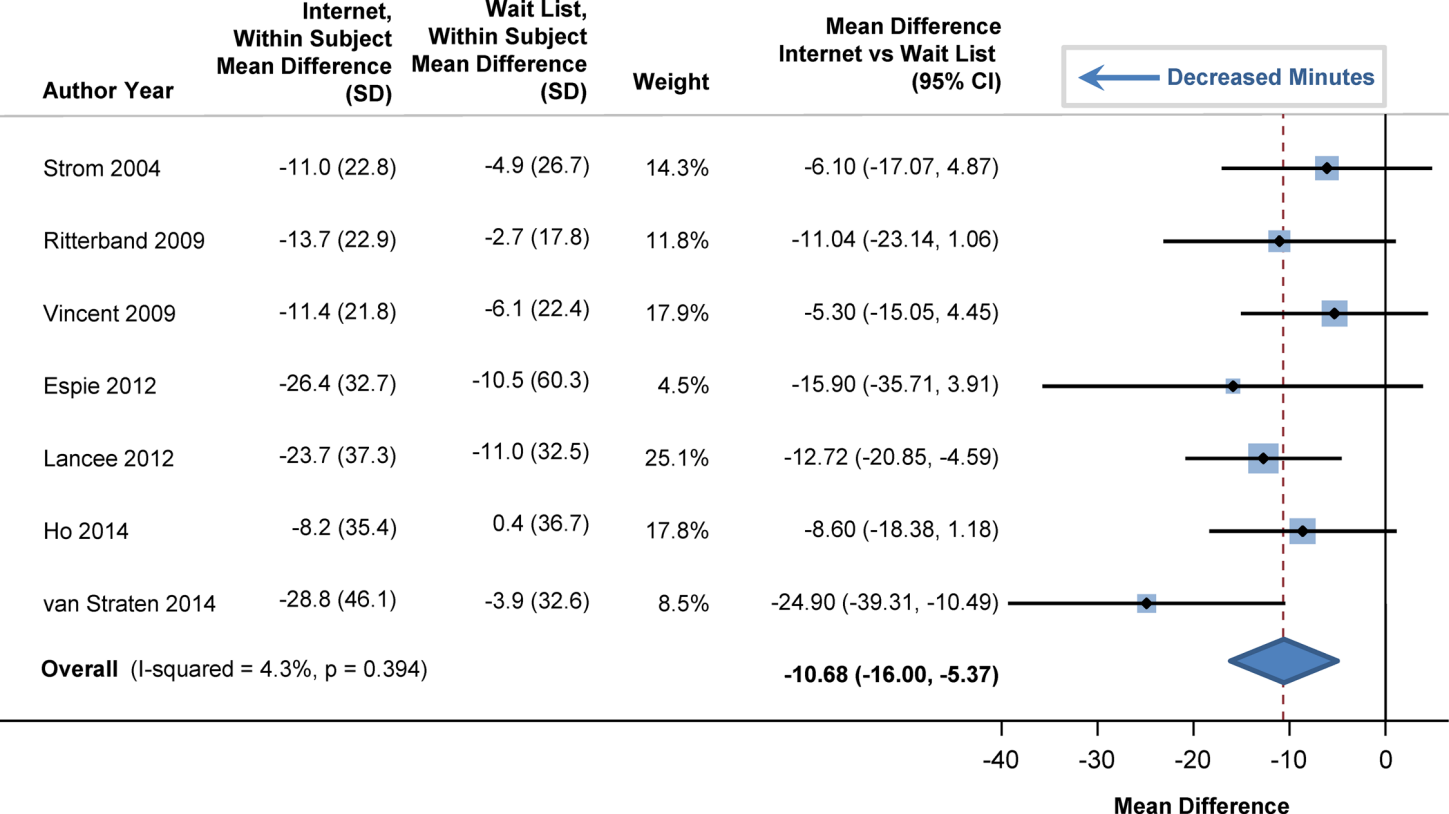
Mean Differences in Sleep Onset Latency between Internet-Delivered Cognitive Behavioral Therapy for Insomnia and Wait List.

**Fig 5:**
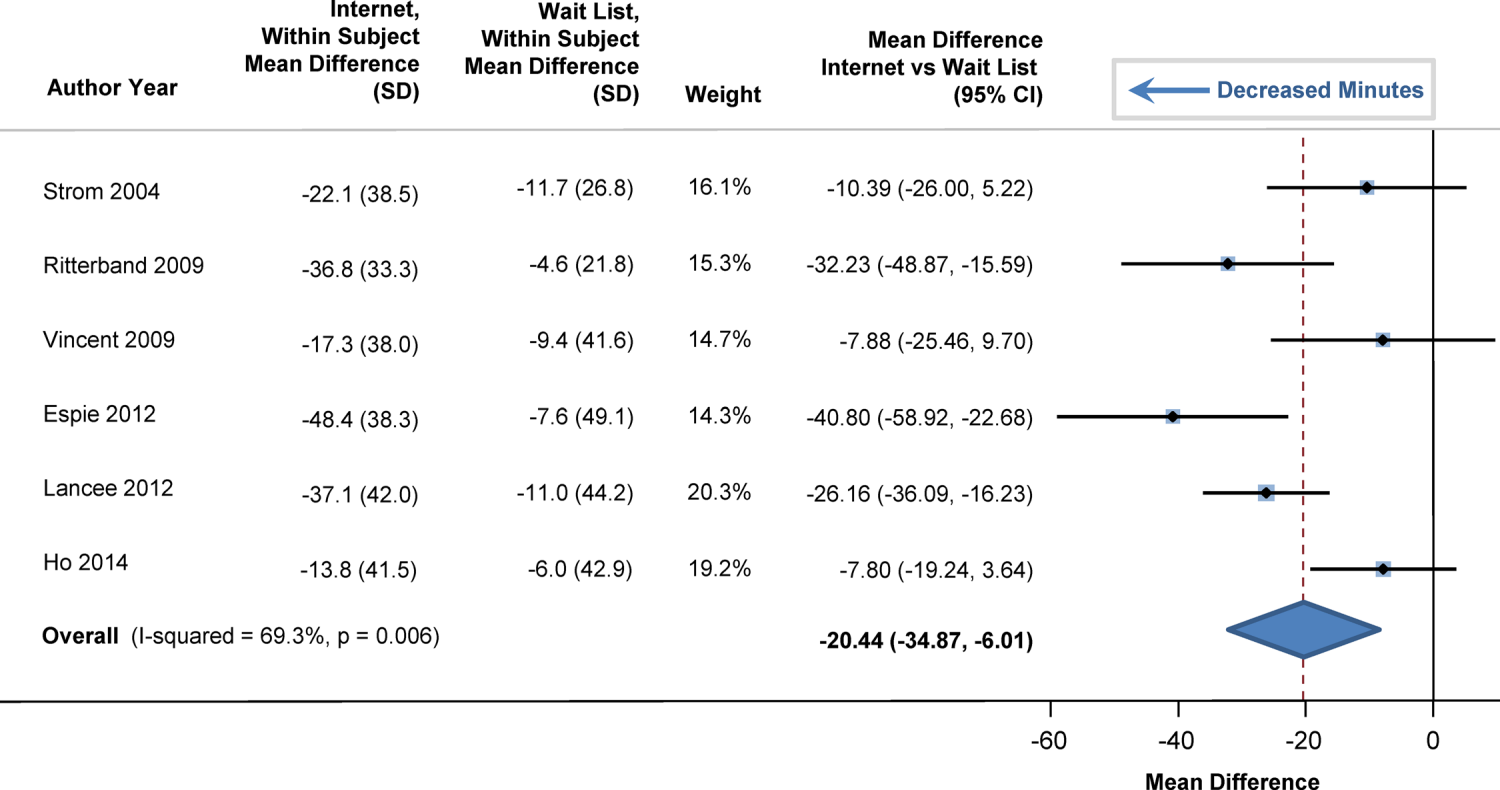
Mean Differences in Wake Time after Sleep Onset between Internet-Delivered Cognitive Behavioral Therapy for Insomnia and Wait List.

**Fig 6:**
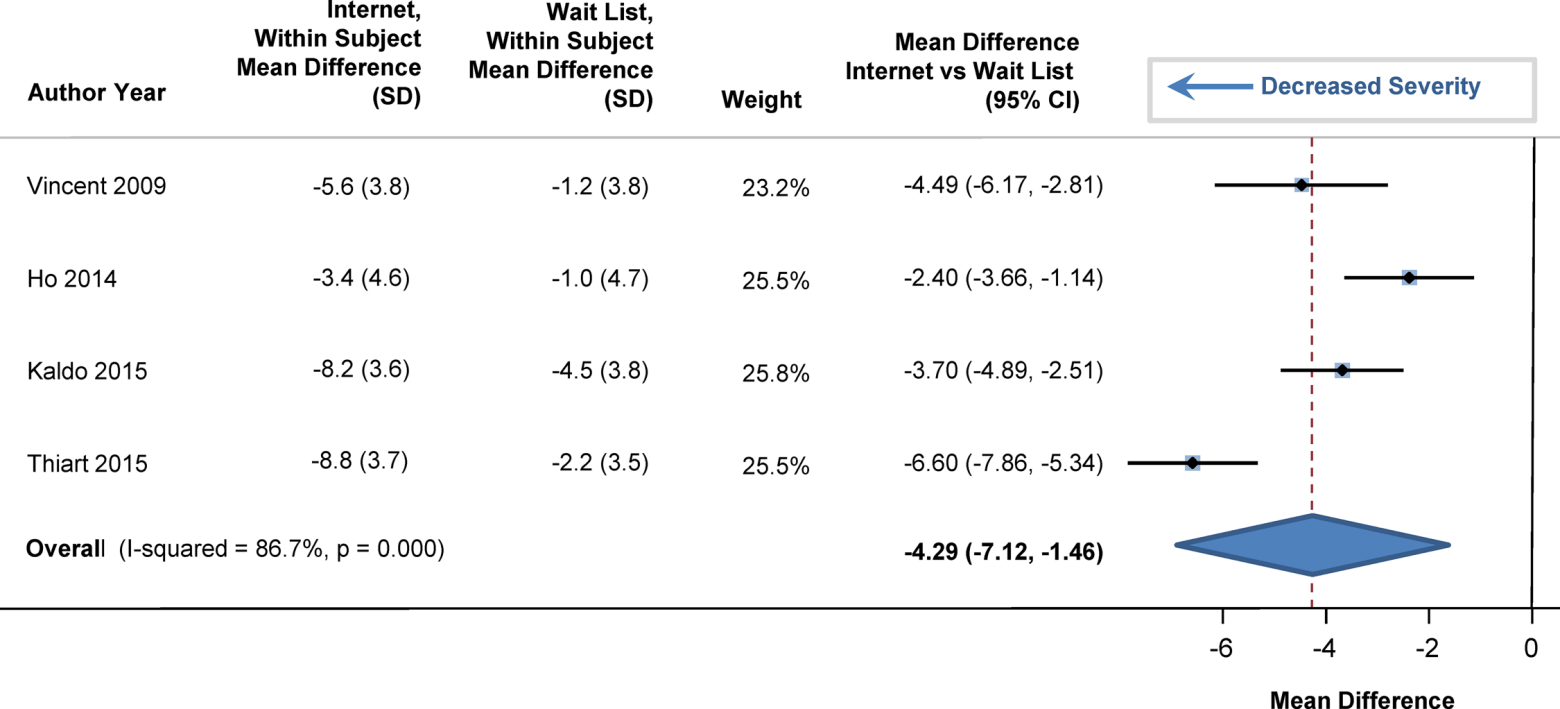
Mean Differences in Insomnia Severity Index between Internet-Delivered Cognitive Behavioral Therapy for Insomnia and Wait List.

At baseline, sleep efficiency was 72% in the participants (95% CI: 69%, 75%). Sleep efficiency significantly improved with internet-delivered CBTI versus a wait list. The pooled estimate for improvement was 7.22% (95% CI: 5.13%, 9.32%; p<0.001), as shown in the forest plot in Fig 2 (I^2^ = 39.5%). At baseline, total sleep time averaged 344 minutes (95% CI: 331, 358), or 5.7 hours. Total sleep time significantly increased by 20 minutes in those who used internet-delivered CBTI vs. control (95% CI: 9 minutes, 31 minutes; p = 0.004) as shown in the forest plot in Fig 3. At baseline, mean sleep onset latency was 48 minutes (95% CI: 41, 56 minutes). As shown in Fig 4, sleep onset latency significantly decreased by 11 minutes in those individuals who received internet-delivered CBTI compared to controls (95% CI: -16, -5 minutes; p = 0.003). At baseline, the mean number of minutes awake after falling asleep (wake time after sleep onset) was 69 minutes (95% CI: 59, 79 minutes). After participating in the internet-delivered CBTI, there was a 20 minute decrease in wake time after sleep onset compared to controls (95% CI: -35, -6; p = 0.015). The forest plot is shown in Fig 5. At baseline, the insomnia severity index was 17 (95% CI: 16, 18) suggestive of clinical insomnia of moderate severity. After the use of the internet-delivered CBTI, there was a significant decrease in the severity of insomnia by 4.29 points (95% CI: -7.12, -1.46; p = 0.017) compared to control (Fig 6).

**Fig 7:**
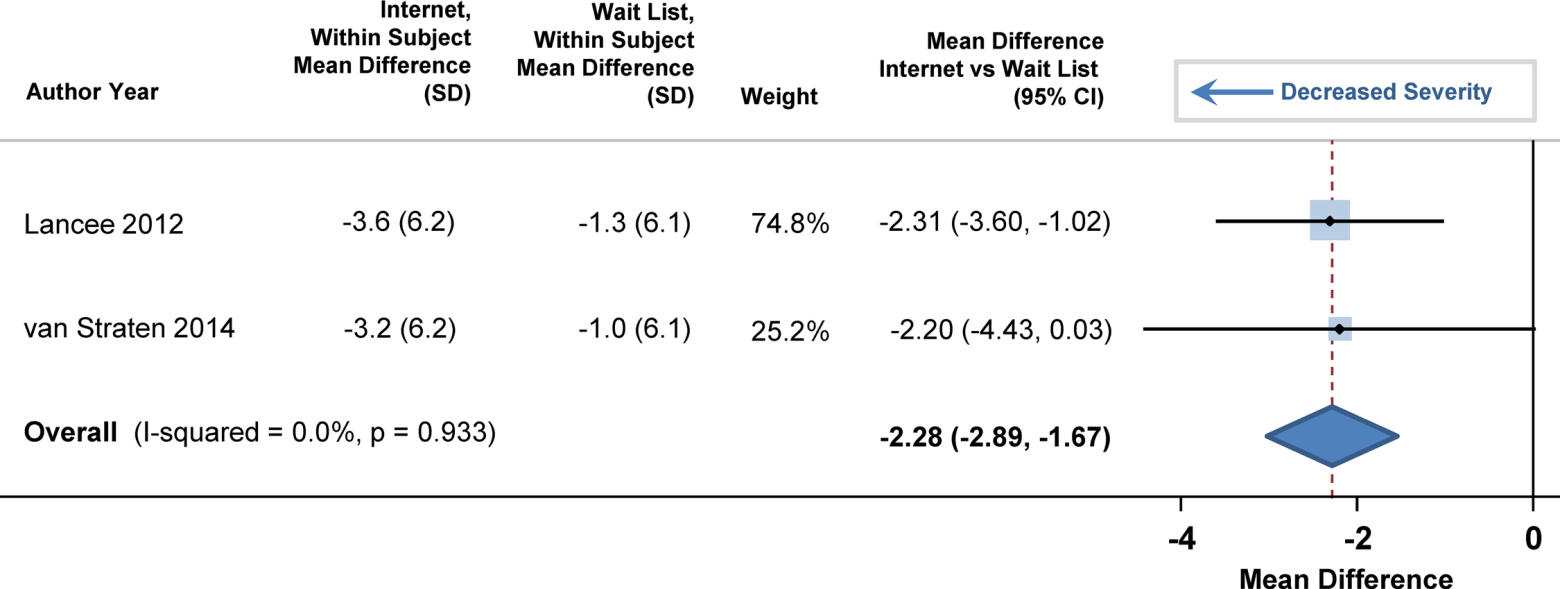
Mean Differences in Depression between Internet-Delivered Cognitive Behavioral Therapy for Insomnia and Wait List.

At baseline, the mean score on the Center for Epidemiologic Studies Depression Scale was 13.1 (95% CI: 11.5, 14.6) in the participants, with higher scores indicating more severe depression. The pooled mean difference in depression scores significantly decreased by 2.28 points (95% CI: -2.89, -1.67; p = 0.013) in individuals who received the internet-delivered CBTI compared to persons who were on a wait list (Fig 7).

Some sleep measures were not statistically different between internet-delivered CBTI and a wait list. For example, the mean Pittsburgh Sleep Quality Index was 10 (95% CI, 7, 12) at baseline, indicating poor sleep quality. After the use of internet-delivered CBTI, the pooled estimate was -2.3 (95% CI: -4.8, 0.3; p = 0.06) compared to controls. At baseline, the time spent in bed was 477 minutes (95% CI: 456, 498) or approximately 8 hours. The pooled estimate comparing internet-delivered CBTI with wait list versus controls was -6.3 minutes (95% CI: -20.2, 7.6; p = 0.19), indicating no significant decrease in the time spent in bed. At baseline, the mean number of nocturnal awakenings was 2.3 (95% CI: 2.1, 2.6). The pooled estimate for the difference in number of nocturnal awakenings in the internet-delivered CBTI group versus wait list was -0.3 (95% CI: -7, 0.1; p = 0.09). Sleep quality was measured on a 5-point scale with an increase indicating better quality. At baseline, the mean sleep quality was 2.9 (95% CI: 1.6, 4.1). The pooled estimate comparing internet-delivered CBTI versus wait list was 0.4 (95% CI: -0.1, 0.8; p = 0.09) indicating no statistically significant improvement. There were 2 trials with different sleep quality measures. Espie and colleagues reported sleep quality on a 100-point scale and found that internet-delivered CBTI significantly improved sleep quality relative to usual care (p<0.001) [26]. Ho and colleagues also reported a significant improvement in sleep quality (p = 0.01) with internet-delivered CBTI compared to a wait list [27].

### Long Term Follow-up

**Fig 8:**
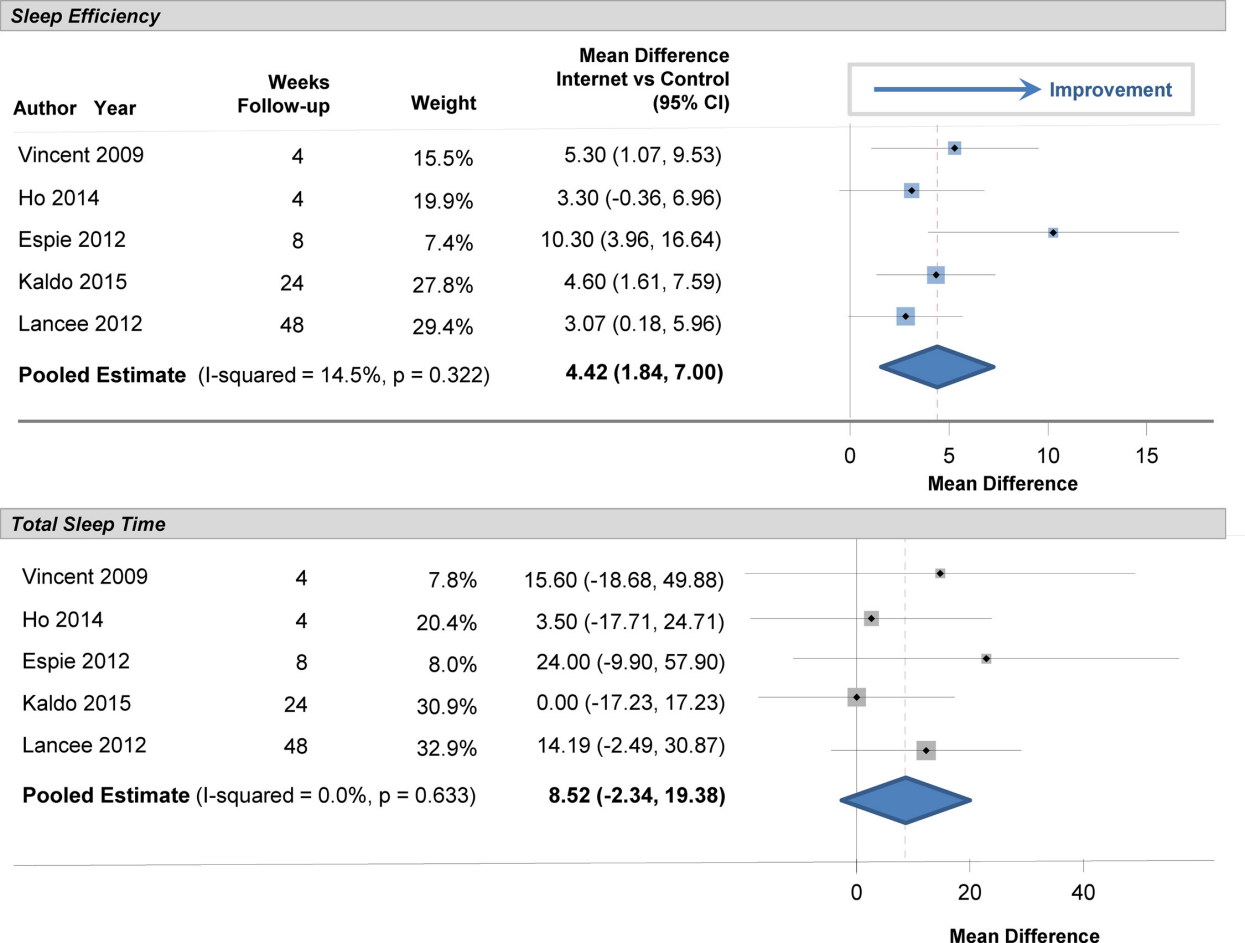
Mean Differences in Sleep Measures between Internet-Delivered Cognitive Behavioral Therapy for Insomnia and Control after Long-Term Follow-up.

**Fig 9:**
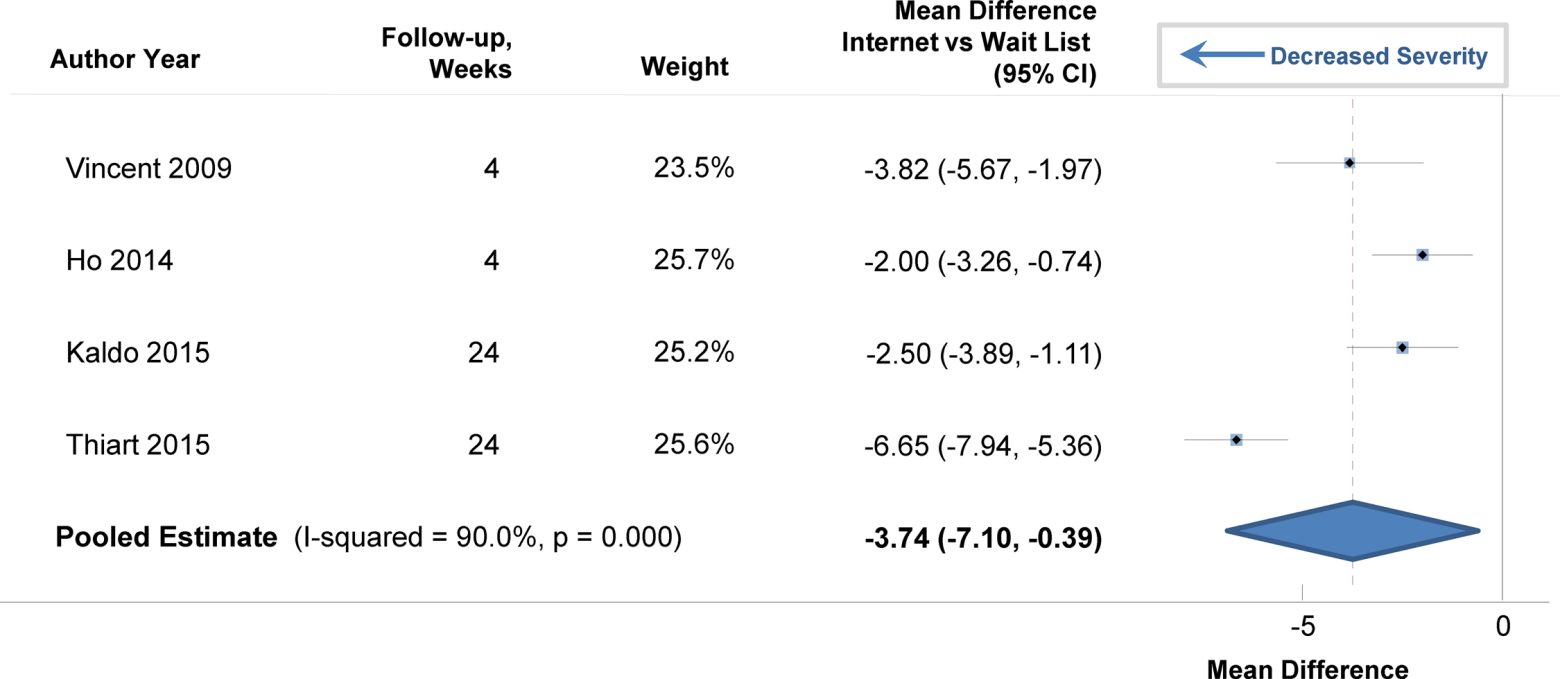
Mean Differences in Insomnia Severity Index between Internet-Delivered Cognitive Behavioral Therapy for Insomnia and Wait List after Long-Term Follow-up.

There were several randomized trials that included data from long-term follow-up after the post-test measurement of sleep measures. Improvement in sleep efficiency with internet-delivered CBTI remained statistically significant after long-term follow-up (Fig 8). The pooled estimate was 4.4% (95% CI: 1.8%, 7.0%; p = 0.009). The pooled estimate for the improvement in total sleep time was not statistically significant (8.5 minutes; 95% CI: -2, 19.3 minutes; p = 0.095). However, the insomnia severity index decreased to a greater degree over the long-term in persons who used internet-delivered CBTI than in those in the control group (Fig 9). The pooled estimate was -3.7 (95% CI: -7.10,-0.39; p = 0.038), indicating an improvement in the severity of insomnia with the internet approach. In one of the trials [25], results from long-term follow-up (48 weeks) were available for depression and the authors found a decrease in the severity of depression with internet-delivered CBTI compared to a waiting list (p<0.001).

### Internet-delivered CBTI versus Other Delivery Methods

**Fig 10:**
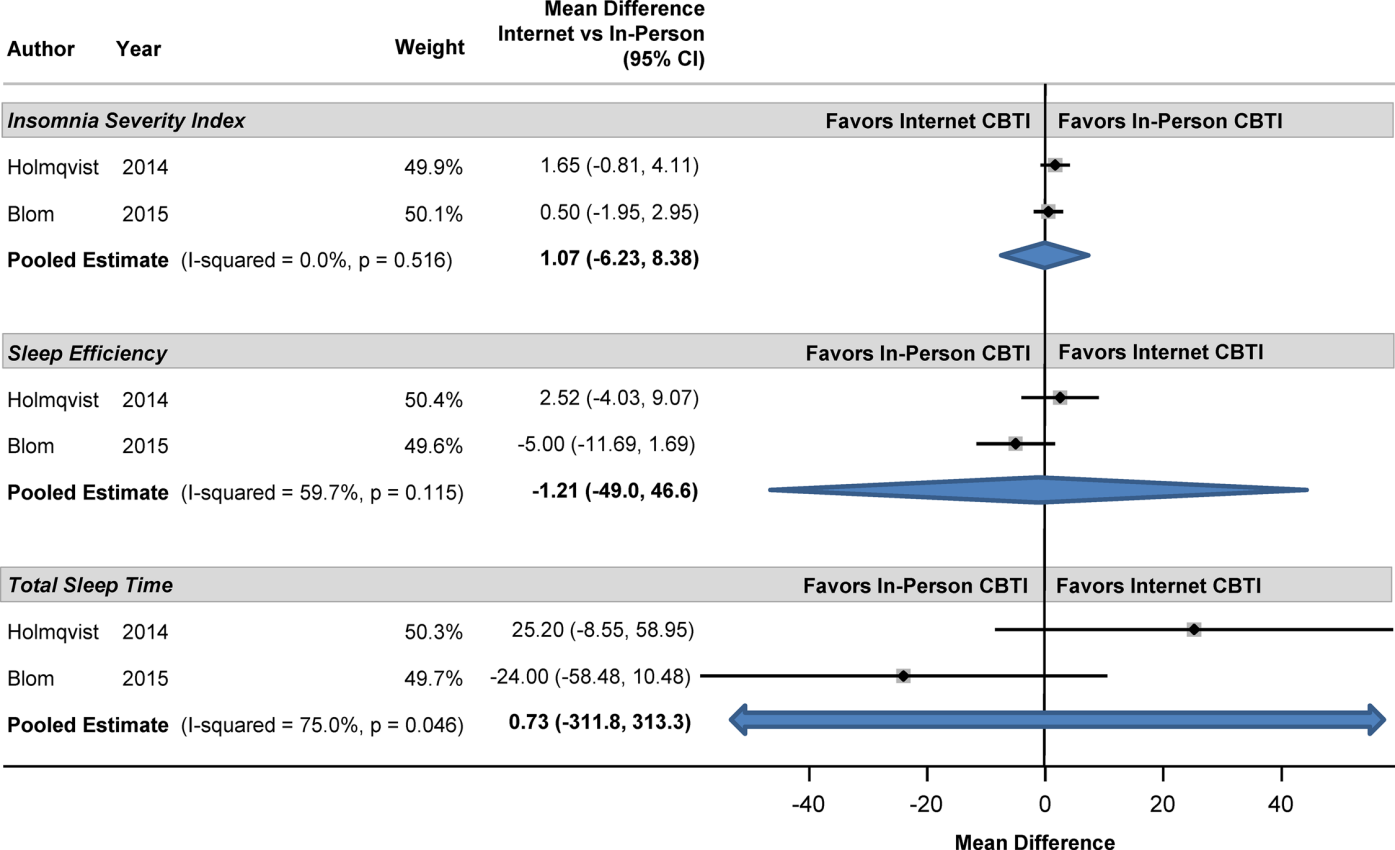
Mean Differences in Sleep Measures between Internet-Delivered and In-Person Cognitive Behavioral Therapy for Insomnia.

Several of the trials compared internet-delivered CBTI to other comparators. For example, Blom [25] and Holmqvist [28] compared internet-delivered to in-person CBTI. As shown in the forest plot in Fig 10, there were no statistically significant differences between these two methods of CBTI delivery. In addition, data were available from a randomized trial in the Netherlands which compared internet CBTI with paper-and-pencil CBTI [30]. There were no significant differences between these two approaches on sleep efficiency, sleep onset latency, total sleep time, wake time after sleep onset, number of awakenings, or insomnia severity index. In a Chinese population, Ho and colleagues compared internet-delivered CBTI to internet-delivered CBTI with a supplementary 15-minute weekly phone call from a psychologist [27]. These two approaches were equivalent (p>0.05) in terms of sleep efficiency, sleep onset latency, sleep quality, insomnia severity index, and severity of anxiety and depression for both the immediate post-intervention period as well as at the 4-week follow-up. However, Lancee compared internet-delivered CBTI to internet-delivered CBTI with supplementary weekly emails in a Dutch population [31]. The approach with weekly emails led to more improved scores on sleep efficiency, sleep onset, wake after sleep onset, number of awakenings, insomnia complaints, depression and anxiety. Blom and colleagues tested internet-delivered CBTI to internet-delivered CBT for depression in patients with both insomnia and depression [24]. At the follow-up period of 12 months, the insomnia severity index improved to a greater degree for those receiving CBT for insomnia than for those receiving CBT for depression. In addition, internet-delivered CBTI led to a decline in the need for insomnia treatment and less use of sleep medication.

### Meta-Regression

Results from meta-regression indicated that age did not modify the association between CBTI and sleep efficiency (p = 0.166) or insomnia severity index (p = 0.473), nor did it significantly affect mean differences for total sleep time (p = 0.562), sleep onset latency (p = 0.310) or wake time after sleep onset (p = 0.188). Similarly, there was no significant effect modification by gender (p = 0.470 for sleep efficiency, p = 0.918 for insomnia severity index, p = 0.192 for total sleep time, p = 0.368 for sleep onset latency, and p = 0.138 for wake time after sleep onset).

### Mechanism of Change

**Fig 11:**
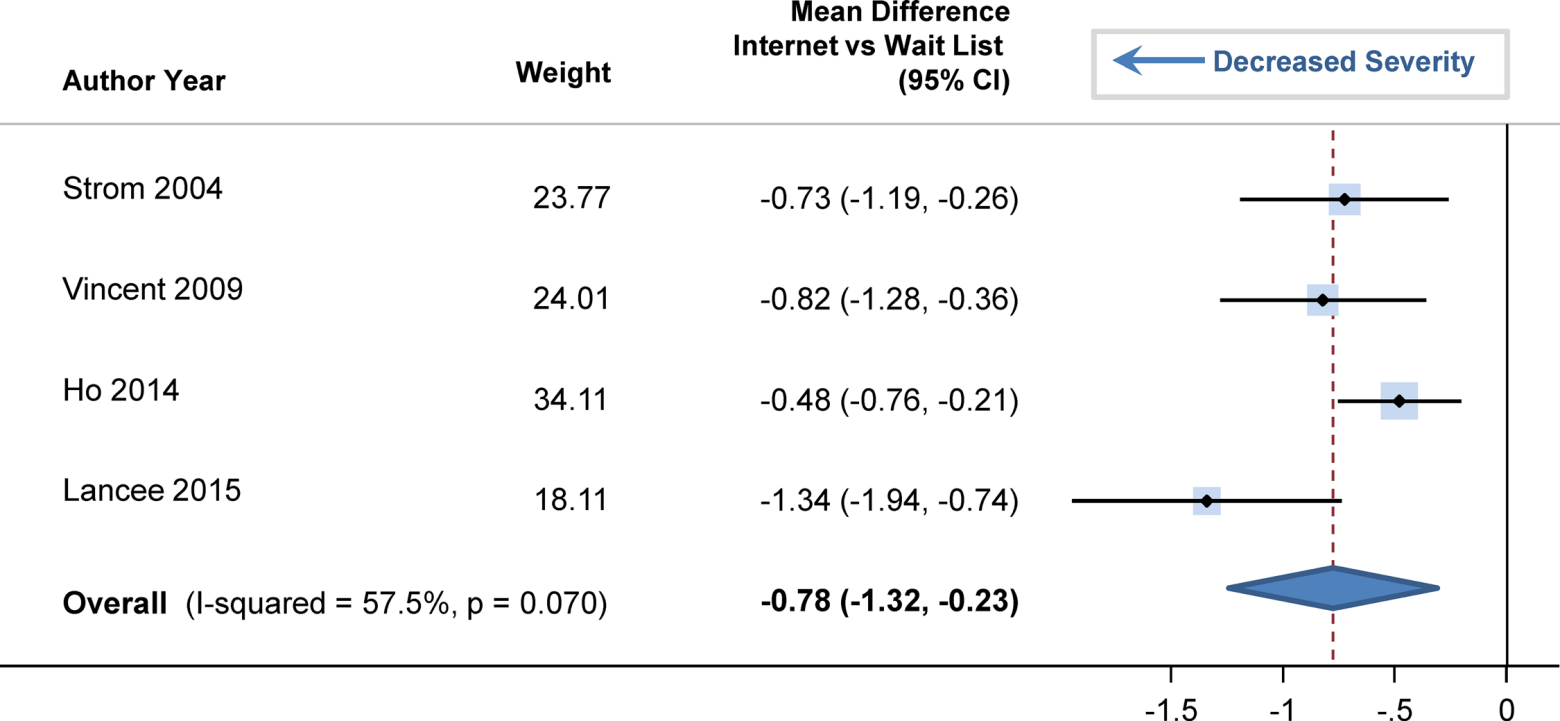
Standardized Mean Differences in Dysfunctional Belief and Attitudes about Sleep, comparing Internet-Delivered Cognitive Behavioral Therapy for Insomnia and Wait List.

There were 4 trials that assessed whether internet-delivered CBTI changed dysfunctional beliefs related to sleep, which are hypothesized to mediate the sleep effects (e.g., “I need 8 hours of sleep to feel refreshed and function well during the day.”) [27,32,35,38]. There was a significant decrease in dysfunctional beliefs through internet-delivered CBTI versus a wait list (Fig 11). The pooled standardized mean difference was -0.78 (95% CI: -1.32, -0.23; p = 0.020). There was only 1 trial that evaluated sleep-related behaviors from the Sleep-Related Behaviors Questionnaire [32]. The authors found that sleep-related behaviors (such as watching the clock) also significantly mediated the effects of internet-delivered CBTI on insomnia severity–even to greater extent than dysfunctional beliefs [32].

### Risk of Bias

Quality metrics of the trials are listed in Table 3. All trials had robust study designs although concealment of randomization was not uniformly stated. Eleven of the trials included at least one negative control group, the most common being a wait list. Most statistical analyses were conducted as intent-to-treat. Owing to the nature of the intervention (internet use), blinding of the participants was not possible and sleep measures were assessed and recorded by participants (e.g., quality of sleep). For all 15 trials, sleep diaries were used to obtain time-related measures (e.g., total sleep time, time in bed) and online questionnaires were used to obtain patient-centered indices (Insomnia Severity Index, Pittsburgh Sleep Quality Index, sleep quality). The sleep measures were standard instruments utilized in sleep research and have been validated (including through web-based delivery) [39–41]. There was no evidence of publication bias (sleep efficiency, p = 0.511; total sleep time, p = 0.962; sleep onset latency, p = 0.318; wake time after sleep onset, p = 0.802; insomnia severity index, p = 0.892).

**Table 3:** Study Quality Assessment.

Author	Year	Concurrent Between-Person Comparator	Within-Person Comparison	Random Sequence Generation	Allocation Concealment	Type of Negative Control	Intent-to-Treat Analyses	Incomplete Outcome Data
Blom[24]	2015	Yes	Yes	Computer	Yes	None	Yes	Low risk
Blom[25]	2015	Yes	Yes	Computer	Yes	None	Yes	Low risk
Espie[26]	2012	Yes	Yes	Computer	Yes	Treatment as usual	Yes	Low risk
Ho[27]	2014	Yes	Yes	Computer	Yes	Wait list	Yes	Low risk
Holmqvist[28]	2014	Yes	Yes	Computer	Not stated	None	No	Low risk
Kaldo[29]	2015	Yes	Yes	Computer	Yes	Internet non-CBTI control	Yes	Low risk
Lancee[30]	2012	Yes	Yes	Computer	Not stated	Wait list	Yes	Low risk
Lancee[31]	2013	Yes	Yes	Computer	Not stated	None	Yes	Low risk
Lancee[32]	2015	Yes	Yes	Not stated	Not stated	Wait list	Yes	Low risk
Morris[33]	2015	Yes	Yes	Computer	No	Wait list	Yes	Low risk
Ritterband[34]	2009	Yes	Yes	Computer	No	Wait list	Yes	Low risk
Strom[35]	2004	Yes	Yes	Not stated	Not stated	Wait list	No	Low risk
Thiart[36]	2015	Yes	Yes	Not stated	Not stated	Wait list	Yes	Low risk
van Straten[37]	2014	Yes	Yes	Computer	Yes	Wait list	Yes	Low risk
Vincent[38]	2009	Yes	Yes	Random numbers table	No	Wait list	No	Low risk

In the internet-delivered CBTI group, 74.5% completed the intervention (95% CI: 64.5%, 83.4%). This compared with 80.3% in the wait list control (95% CI: 73.7%, 86.3%). There was no significant difference between these two completion rates (p = 0.198).

## Discussion

In this systematic review and meta-analysis, internet-delivered CBTI was effective in improving sleep efficiency, insomnia severity, total sleep time, sleep onset latency, and wake time after sleep onset. Moreover, an improvement in sleep efficacy and a decline in the severity of insomnia were maintained over a long follow-up period. It is important to note that the degree of improvement in the insomnia severity index was not only statistically significant, but also clinically meaningful: at baseline, the participants had, on average, clinical insomnia of moderate severity. The drop in the index of 4.29 points brought the participants, on average, to a lower clinical level (sub-threshold insomnia). In addition, internet-delivered CBTI significantly decreased the severity of depressive symptoms. Significant mediators of this improvement were found to be changes in dysfunctional beliefs and sleep-related behaviors.

We found that internet-delivered CBTI had similar efficacy as in-person delivery by therapists. It also had similar efficacy as CBTI delivered through printed materials (paper and pencil). Enhancement of internet-delivered CBTI through weekly phone calls from a therapist did not significantly improve sleep, but (in one study), enhancement of the internet approach with supplemental emails from therapists did. Collectively, these findings suggest promise for this modality of CBTI as an initial avenue for treatment of insomnia because of its accessibility. There are now more than 3 billion users of the Internet worldwide [42] and, when online treatments are found to be effective, this portal may provide an accessible avenue to improve health.

There have been at least two other systematic reviews published on this topic [12,13]. Cheng and Dizon reviewed the evidence from 6 randomized trials on computerized CBT but not all studies included an internet platform [12]. There was overlap of 3 trials with our review due to differences in eligibility requirements, but the results were similar. They found a significant improvement in sleep quality, sleep efficiency, the number of awakenings, sleep onset latency and severity of insomnia. Zachariae and colleagues reviewed evidence from 11 randomized trials (1460 participants) and also reported similar results [13]. They found improvement in severity of insomnia, sleep efficiency, sleep quality, wake after sleep onset, sleep onset latency, total sleep time and number of nocturnal awakenings. There was an overlap of 8 trials with our study due to differences in eligibility. Our review of 15 randomized trials with 2392 participants employed slightly different analytic methods in that the Hartung-Knapp-Sidak-Jonkman random effects model is more conservative but recommended above the fixed models used in the Cheng review and the DerSimonian-Laird random effects model [21,22]. Moreover, previous reviews used the between-group mean difference only [12] or the within-group mean difference only [13] to calculate effect sizes. Since the 15 trials in our review all utilized a pretest-posttest control group design, we used both the within- and between-group comparisons to calculate the final effect. This is slightly more conservative but more closely matches the design of the trials and the totality of the underlying comparisons.

Dissemination of online mental health services is becoming increasingly available and has the potential of revolutionizing health care delivery [43]. For example, meta-analyses of randomized trials have reported that online treatments for depression, anxiety, alcohol use and tobacco use are effective [43]. Internet-based mental health platforms are already approved by the National Health Service in the United Kingdom as part of a stepped care approach, with internet-delivered CBTI recommended as one of the options for individuals with sub-threshold symptoms and those with certain mental health disorders [44].

Since persons with various mental disorders often experience insomnia, access to internet-delivered CBTI may provide flexibility and convenience (e.g., in those that suffer social anxiety or agoraphobia). Additionally, internet-delivered CBTI holds the potential to improve access to insomnia treatment for individuals with limited transportation options or those that live in rural areas and cannot physically access a trained therapist. Furthermore, psychiatric patients may experience stigma when having to seek assistance for mental health needs. An online CBTI program may be valuable as it provides a less stigmatized first-step entry to mental health services, whereby individuals can simultaneously improve their sleep and mental health without having to focus only on psychiatric symptoms. Links to in-person assistance could be made available for those desiring additional services.

### Limitations

Most of the randomized trials were conducted in Europe with only 3 in North America and 1 in Asia. Therefore, additional trials in other populations may be warranted. The majority of the trials were conducted in middle-aged adults with only one study in young adults [33], so randomized trials in young and older adults may provide clarification as to whether there are age-specific effects of internet-delivered CBTI. Additional trials that more fully delineate the most effective features of internet engagement may be informative, as well as whether supplemental aids or assistance would provide benefits. Future studies could also be improved by providing more consistent lengths of follow-up for the evaluation of the sustainability of the effects. In addition, participants generally suffered from insomnia of moderate severity so further studies are needed to evaluate internet-delivered CBTI for those with more severe insomnia.

Trials targeted to specific groups at higher risk of insomnia may be informative. Insomnia occurs in 1 of every 4 U.S. workers, so trials utilizing internet-delivered CBTI may be prudent, particularly in those doing shift work [45,46]. Another high-risk group are veterans in whom insomnia can exacerbate other mental health disorders such as depression, post-traumatic stress disorder, and increases the risk of suicide [47,48].

It could be argued that such trials do not address a possible placebo effect–that the use of the Internet itself could have a positive effect on health. Kaldo and colleagues directly addressed this possibility by using an internet control that did not contain CBTI [29]. Their control included abbreviated education regarding sleep hygiene, relaxation, general stress management, and mindfulness but did not include sleep restriction, stimulus control, or directed advice on the management of sleep patterns. They found a significant increase in sleep efficiency, as well as a significant decrease in the severity of insomnia by using internet-delivered CBTI versus an internet control.

While there was an improvement in sleep efficiency and the severity of insomnia, not all sleep measures showed improvement. For example, time in bed and the number of nocturnal awakenings were not significant although each point estimate in the trials trended towards improvement. There were slight variations in the CBTI programs across the trials and future evaluations could target which elements of the programs had the greatest impact. Moreover, self-reported sleep diaries were utilized to record sleep patterns, so future studies could potentially improve measurements by utilizing available technologies such as actigraphy for time-relevant measures. The current results, however, suggest that sleep efficiency and the severity of insomnia can be improved, even without changes in the amount of time in bed.

It should be noted that not all subjects in the randomized controlled trials completed the study (74.5% in the intervention group versus 80.3% in the control), although most trials utilized an intent-to-treat analysis. There was no significant difference between completion rates in the intervention versus control groups. Adverse effects were not specifically mentioned in the trials although no adverse effects of CBTI were reported for trials that utilized face-to-face therapist guided CBTI [11]. A component of CBTI (sleep restriction therapy) was reported to have side-effects such as fatigue, extreme sleepiness, and reduced motivation [49]. Unfortunately, this study did not contain a control group (individuals with insomnia who did not receive sleep restriction therapy) and therefore, it is unknown whether these symptoms were due to the underlying insomnia disorder itself or this specific treatment [49]. In a similar study, side effects of sleep restriction therapy were found during the course of treatment (again, without a patient control group) even though both sleep efficiency and insomnia severity significantly improved over time for those receiving this therapy [50]. For future studies using CBTI, it is important that any adverse effects are measured (similar to any other randomized controlled trial) and that proper controls with insomnia are utilized so that the background rate of effects can be compared.

These limitations notwithstanding, our study has important strengths. The most notable is the trial design. Unlike most randomized trials that include a between-group comparator, trials of internet-delivered CBTI have the added component of the within-person comparison which is a powerful controller of confounding. That is, when there is an improvement in sleep efficiency over time in a given person, this change is not due to confounding by gender, race, genetic profile or past history of medical conditions because it is measured in the same person (i.e., such potential confounders are held constant). This “difference in differences” approach is often used in econometrics [51]. Therefore, all of the trials in our review not only contained randomization (thus eliminating selection bias) but also addressed potential confounding to a greater degree than just randomization alone.

Given the burden of insomnia on population health and wellbeing, these findings have important policy implications. While CBT is covered under some health insurance plans, it is unclear whether internet-delivery of CBTI, in particular, is covered. There are several options that could be considered. One option would be to have the Centers for Disease Control and Prevention, the World Health Organization, or another widely-recognized health agency host an online CBTI program with links to additional resources when contact with physicians and trained therapists is desired. Another option would be for health insurers to include internet-delivered CBTI within their existing disease management programs. Employers may also consider offering reimbursement or incentives for internet-delivered CBTI to their employees, since insomnia does impact productivity [46].

In conclusion, evidence from randomized controlled trials indicates that internet-delivered cognitive behavioral therapy for insomnia is effective. Now is the time for an international initiative to educate the public on the availability of this treatment and to provide avenues for access.

## Supporting Information

S1 Dataset Data File. (XLS)

S1 File PRISMA Checklist. (PDF)

S2 File Search Strategy. (PDF)
