# Supplementary material for: Internet-Delivered Cognitive Behavioral Therapy to Treat Insomnia: A Systematic Review and Meta-Analysis
Source: PLoS One. 2016 Feb 11;11(2):e0149139. doi: 10.1371/journal.pone.0149139 (PMC4750912; doi:10.1371/journal.pone.0149139)
Supplement: S2 File — (PDF) [file pone.0149139.s003.pdf]

S2 File. Search strategy

PubMed

("Randomized Controlled Trials as Topic"[Mesh] OR "Randomized Controlled Trial" [Publication Type] OR random*) AND (cognitive therapy [mh:noexp] OR cbti[tiab] OR cbt-i[tiab] OR "cognitive behaviour" [tw] OR "cognitive behavior" [tw] OR "cognitive behavioural" [tw] OR "cognitive behavioral" [tw]) AND (sleep initiation and maintenance disorders [mh] OR insomnia [tw]) AND (internet [mh] or internet [tiab] OR web [tiab] OR "web-based" [tiab] OR computer [tiab] OR online [tiab])

CINAHL

MH (MH "Cognitive Therapy+") OR TI (cbt-i OR cbti OR "cognitive behavioral" OR "cognitive behavioural" OR "cognitive behavior" OR "cognitive behaviour") OR AB (cbt-i OR cbti OR "cognitive behavioral" OR "cognitive behavioural" OR "cognitive behavior" OR "cognitive behaviour")

AND

TI (web OR internet OR computer OR web-based OR online) OR AB (web OR internet OR computer OR web-based OR internet OR online)

AND

MH (MH "Insomnia") OR TI insomnia OR AB insomnia

AND

AB random* OR TI random*

Embase

'insomnia'/exp OR insomnia:ab,ti AND 'cognitive therapy'/exp OR cognitive NEXT/1 behavior* OR cognitive NEXT/1 behavi?r* AND web:ab,ti OR internet:ab,ti OR computer:ab,ti OR online:ab,ti OR 'web based':ab,ti AND random*:ab,ti

PsycInfo

TX insomnia AND TX ("cognitive behavior" OR "cognitive behaviour" OR "cognitive behavioural" OR "cognitive behavioral") AND TX (web OR internet OR computer OR web-based OR online) AND TX random*

CENTRAL

'insomnia in Title, Abstract, Keywords and "cognitive behavior" OR "cognitive behaviour" OR "cognitive behavioural" OR "cognitive behavioral" in Title, Abstract, Keywords and web OR internet OR computer OR web-based OR online in Title, Abstract, Keywords and random* in Keywords in Trials'

WoS

TOPIC: (insomnia) *AND* **TOPIC:** ("cognitive behavior" OR "cognitive behavioural" OR "cognitive behavioral" OR "cognitive behaviour") *AND* **TOPIC:**(web OR Internet OR computer OR web-based OR online) *AND* **TOPIC:** (random*)

Indexes=SCI-EXPANDED, CPCI-S Timespan=All years
